# Differences in time course activation of dorsolateral prefrontal cortex associated with low or high risk choices in a gambling task

**DOI:** 10.3389/fnhum.2014.00464

**Published:** 2014-06-24

**Authors:** Stefano Bembich, Andrea Clarici, Cristina Vecchiet, Giulio Baldassi, Gabriele Cont, Sergio Demarini

**Affiliations:** ^1^Institute for Maternal and Child Health, IRCCS “Burlo Garofolo”Trieste, Italy; ^2^Psychiatric Clinic Unit, Department of Medical, Surgical and Health Sciences, University of TriesteTrieste, Italy

**Keywords:** multichannel NIRS, DLPFC, risk, Iowa Gambling Task, attention shifting, response inhibition

## Abstract

Prefrontal cortex plays an important role in decision making (DM), supporting choices in the ordinary uncertainty of everyday life. To assess DM in an unpredictable situation, a playing card task, such as the Iowa Gambling Task (IGT), has been proposed. This task is supposed to specifically test emotion-based learning, linked to the integrity of the ventromedial prefrontal cortex (VMPFC). However, the dorsolateral prefrontal cortex (DLPFC) has demonstrated a role in IGT performance too. Our aim was to study, by multichannel near-infrared spectroscopy, the contribution of DLPFC to the IGT execution over time. We tested the hypothesis that low and high risk choices would differentially activate DLPFC, as IGT execution progressed. We enrolled 11 healthy adults. To identify DLPFC activation associated with IGT choices, we compared regional differences in oxy-hemoglobin variation, from baseline to the event. The time course of task execution was divided in four periods, each one consisting of 25 choices, and DLPFC activation was distinctly analyzed for low and high risk choices in each period. We found different time courses in DLPFC activation, associated with low or high risk choices. During the first period, a significant DLPFC activation emerged with low risk choices, whereas, during the second period, we found a cortical activation with high risk choices. Then, DLPFC activation decreased to non-significant levels during the third and fourth period. This study shows that DLPFC involvement in IGT execution is differentiated over time and according to choice risk level. DLPFC is activated only in the first half of the task, earlier by low risk and later by high risk choices. We speculate that DLPFC may sustain initial and more cognitive functions, such as attention shifting and response inhibition. The lack of DLPFC activation, as the task progresses, may be due to VMPFC activation, not detectable by fNIRS, which takes over the IGT execution in its second half.

## Introduction

There is a general agreement in the role of the prefrontal cortex in decision-making (DM), under conditions of complexity and unpredictability (e.g., Bechara et al., 2000a; Bechara, 2001; Clark et al., 2003; Wise, 2008; Gläscher et al., 2012). According to Damasio's “somatic marker hypothesis” (Damasio, 1994), patients with ventromedial prefrontal cortex (VMPFC) damage develop severe impairments in personal and social DM, while other intellectual abilities are usually well preserved. There appears to be an important deficit in the emotional learning process: the somatic activation of an emotional response is no longer associated with DM, in uncertain conditions, therefore impairing the DM process itself.

In order to assess the complex nature of human DM under uncertainty, the Iowa Gambling Task (IGT) has been developed (Bechara et al., 1994). It is a playing card task which simulates real-life decisions. The IGT is supposed to specifically test emotion-based learning, linked to DM under uncertainty. For example, during the task, participants show an anticipatory skin conductance response associated with risky choices (Bechara et al., 1999), while patients with a damage in VMPFC, both fail the test and do not show anticipatory skin conductance responses (Bechara et al., 1994, 2000b). Additionally, VMPFC may play a role in the continuous updating of expectations about reward and punishment based on experience (Fellows and Farah, 2005; Oya et al., 2005; Stocco and Fum, 2007).

Other areas of the prefrontal cortex may play a relevant role in DM processes associated with the IGT execution. Among these, the most important seems to be the dorsolateral prefrontal cortex (DLPFC), whose role in a gambling task has been demonstrated both by clinical and neuroimaging studies. Patients with a lesion in the right DLPFC showed significantly worse performances than patients with focal lesions in other areas (with the exclusion of VMPFC) or controls (e.g., Manes et al., 2002; Clark et al., 2003). Moreover, Fellows and Farah (2005) found that eliminating the need for a reversal learning from IGT improved the performance of VMPFC patients, but not that of DLPFC patients. In addition, using positron emission tomography (PET), a significant activation in right DLPFC was found during IGT execution (e.g., Ernst et al., 2002, 2003; Bolla et al., 2003, 2005).

In a recent functional magnetic resonance imaging (fMRI) research, a modified double deck version of IGT was used to study the role of prefrontal cortex in task rule learning (Hartstra et al., 2010). A differential involvement of DLPFC, anterior cingulated cortex (ACC)/pre-supplementary motor area (pre-SMA) and medial orbital frontal cortex (med-OFC) was observed, as IGT execution progressed. DLPFC and ACC/pre-SMA were more active during the first phase of IGT performance, while med-OFC was more active in later phases. However, differential cortical activation according to different choice risk levels was not studied.

So far IGT has been studied only by fMRI (e.g., Lin et al., 2008; Lawrence et al., 2009) or PET (e.g., Ernst et al., 2002). Consequently, the execution of the original task had to be adapted to the spatial and technical limitations of such devices (e.g., implementing a computerized version of IGT or limiting cerebral activity detection to a restricted part of the test). As a possible alternative, closer to real life situations, multichannel near-infrared spectroscopy (NIRS) may be used (e.g., Cazzell et al., 2012). It permits detection of regional hemoglobin concentration changes in the cortex induced by brain activity with fairly good spatial resolution and very high temporal resolution (Maki et al., 1995; Taga et al., 2003), even if detection is limited to the cortical surface. NIRS cortical monitoring is non-invasive, does not require strict motion restriction during measurements (subjects are not put into a scanner), is safe (Ito et al., 2000) and resistant to movement artifacts. These characteristics make it particularly suitable for research concerning also complex cognitive functions with awake healthy subjects in a relatively unrestricted environment.

Although DLPFC activation during IGT has been observed mainly in the initial phases of the performance (Hartstra et al., 2010), there are no studies assessing if such activation is differentially associated with high- or low-risk choices as the task goes on. The aim of this pilot exploratory study was to further assess, by multichannel NIRS, the dynamic contribution of DLPFC to the IGT execution over time, in healthy adults. We tested the hypothesis that DLPFC would be differentially activated by low- and high-risk choices as the IGT execution progressed.

## Materials and methods

### Participants

A total of 11 participants (seven females), aged between 21 and 38 years (mean: 27.7 ± 5.7), participated to this study. They were all healthy and there was no history of neurological, sensory, psychiatric or addictive disorders. All were right handed, as assessed by the Edinburgh Questionnaire (Oldfield, 1971). Written informed consent was obtained from each participant after full procedural and technical explanation of the experiment. The ethics committee of the Institute for Maternal and Child Health IRCCS “Burlo Garofolo”—Trieste (Italy), where all tests were conducted, approved the research.

### The iowa gambling task

We administered the IGT as proposed in the original study of Bechara et al. (1994), using physical playing cards. Participants chose 100 cards from four decks (A, B, C, D) in any sequence they preferred. There were two advantageous or low risk decks (C and D), and two disadvantageous or high risk decks (A and B). The schedule of rewards and punishments differed in decks: in deck A (disadvantageous/high risk) and deck C (advantageous/low risk) there were smaller but more frequent unpredicted punishments, while in deck B (disadvantageous/high risk) and deck D (advantageous/low risk) punishments were greater but less frequent. The only difference from the original task consisted in the value of rewards and punishments and in the currency (Euros rather than Dollars). Participants were actually given a total amount of € 2000 (fake money) at the beginning of the task. When playing on disadvantageous decks, participants won € 100 for every card turned, but incurred in losses between € 150 and € 1250. When playing with advantageous decks, they won € 50 for every card turned and incurred in losses between € 25 and € 250. If subjects chose all the cards in decks A and B they lost comprehensively € 1000, if they chose all the cards in decks C and D they won comprehensively € 1000. Every win or loss actually modified the amount of money held by the participant, and he/she was invited to consider the amount of fake money possessed, won or lost as real and own money. The aim of the game was to win as much money as possible, and to avoid disadvantageous decks. A total net score of less than 50 advantageous choices is the cut-off value that indicates that participants are not choosing cards advantageously.

### Multichannel NIRS recording

In order to detect cortical activity, we used the Hitachi ETG-100 OT device (Hitachi Medical Corporation, Tokyo, Japan), which is a multichannel NIRS system (Maki et al., 1995) recording simultaneously from 24 channels on the cortex. It emits near-infrared light at two wavelengths, 780 and 830 nm, and the reflected light is sampled once every 100 ms. This device estimates changes in the concentration of oxy-hemoglobin (HbO2) deoxy-hemoglobin and total hemoglobin in response to stimulation in the unit of mM•mm, that is the product of the hemoglobin concentration changes expressed in millimolar and the optical path length expressed in millimeters.

**Figure 1:**
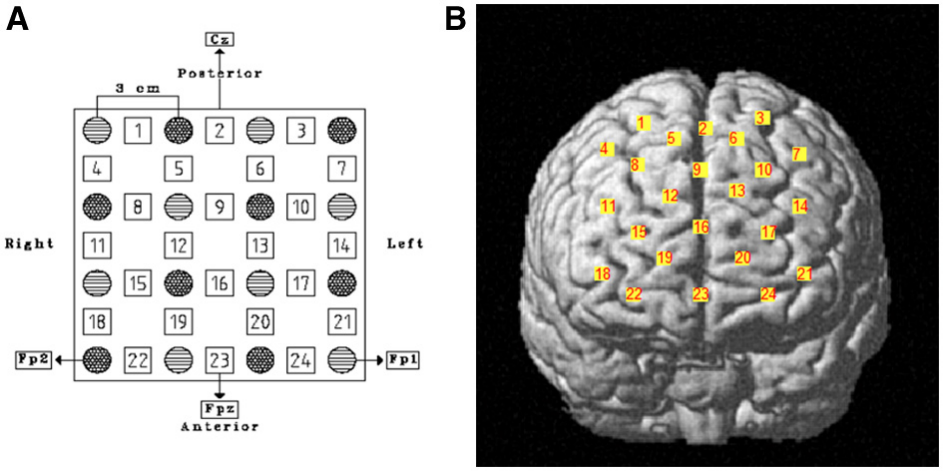
**(A)** Schematic representation of fibers holder positioning over the DLPFC. Stripped circles represent near-infrared emitters and dotted circles detectors. Squares are channels and their respective number. Reference points of the international 10–20 electroencephalography system of electrode placement are indicated as well. **(B)** Channels' projection on DLPFC region of a rendered brain.

For detecting DLPFC activation during the IGT, we used a 4 × 4 plastic fibers holder containing 16 optical fibers (or optodes) of 1 mm in diameter, 8 emitters and 8 detectors, which were placed 3 cm apart and allowed 24 detecting channels. The fibers holder was positioned above the frontal lobes of participants using the international 10–20 EEG placement system (Jasper, 1958). The anterior row of channels (Figure 1) was put on the virtual line joining Fp1 and Fp2 points, placing channel 23 on Fpz. When the holder was positioned, it was made sure that the fibers touched the scalp. The device automatically detects whether the contact is adequate to measure emerging photons.

Cortical areas covered by each channel were identified referring to the methods developed by Tsuzuki et al. (2007) and by Singh et al. (2005), which allow fNIRS data to be probabilistically registered to the standard Montreal Neurological Institute (MNI) space also without using a 3D digitizer, as in our case. The probabilistic localization for each channel is given in Table 1. Cerebral labeling is based on the brain atlas constructed by Tzourio-Mazoyer et al. (2002). To have an estimate of channel projections on a rendered brain, we used the “Spatial registration of NIRS channels location” function of the NIRS-SPM version 4_r1 software (Ye et al., 2009), which is a SPM5 and MATLAB based software package (http://bisp.kaist.ac.kr/NIRS-SPM). Using the “Stand alone” option, not needing MRI images, we obtained a spatial representation of the channel locations on a rendered brain, referring to the MNI coordinates reported in Table 1.

**Table 1:** **Probabilistic cortical channels localization in MNI space and the corresponding cortical labeling**.

**Channel**	**Anatomical label**	**MNI coordinates estimation (mm)**			
		***x***	***y***	***z***	***SD***
1	R F sup gyrus	24	24	60	7.4
2	R/L^*^ F sup medial gyrus	2	28	59	7.9
3	L F sup gyrus	−22	23	61	7.7
4	R F middle gyrus	37	30	50	6.9
5	R F sup gyrus	13	40	54	6.9
6	L F sup gyrus	−12	41	54	6.8
7	L F middle gyrus	−35	30	50	7.2
8	R F sup gyrus	25	47	44	6.1
9	R/L^*^ F sup medial gyrus	2	50	44	6.7
10	L F sup gyrus	−22	47	43	6.7
11	R F middle gyrus	38	52	29	5.6
12	R F sup gyrus	14	61	34	6.1
13	L F sup gyrus	−12	60	35	5.7
14	L F middle gyrus	−36	51	29	6.4
15	R F sup gyrus	26	65	19	4.9
16	R/L^*^ F sup medial gyrus	3	66	22	7.1
17	L F sup gyrus	−24	65	20	5.6
18	R F middle gyrus	39	63	4	4.9
19	R F sup gyrus	15	71	9	4.5
20	L F sup gyrus	−13	72	9	4.9
21	L F middle gyrus	−37	63	4	5.1
22	R F sup orb gyrus	28	68	−5	4.1
23	R/L^*^ F middle orb gyrus	3	68	−4	5.4
24	L F sup orb gyrus	−24	68	−5	4.2

*R, right; L, left; F, frontal; Orb, orbital; sup, superior*.

* *Channels located on the nasion-Inion virtual line*.

## Procedure

**Figure 2:**
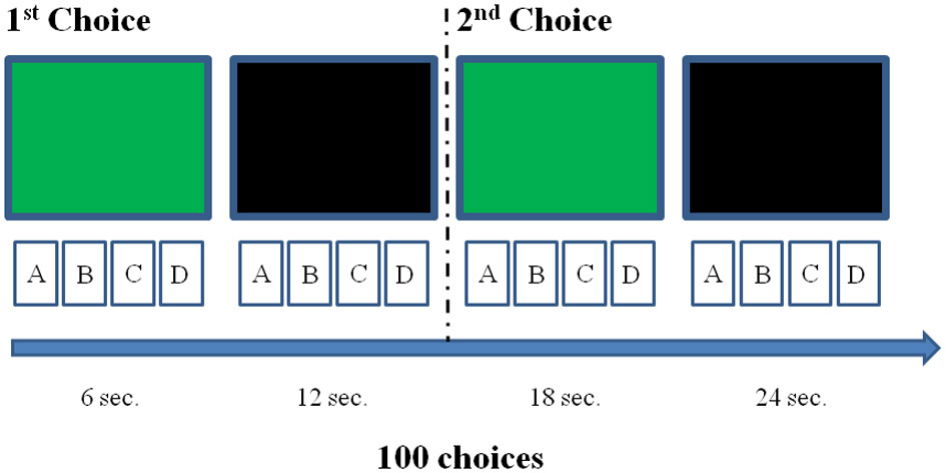
**Illustration of the experimental procedure concerning the first two choices**. Each choice could be made just after the presentation of the green slide, but within 12 s (e.g., the interval between choices we selected to allow the detection of a complete hemodynamic response associated with each decision). The black screen was inserted to fill the 12 s inter-choice interval creating an alternation with the green slide, which had to be again presented to allow the next choice. This was repeated for 100 times.

We administered the IGT in a soft lighted and sound isolated room, and participants sat on a chair in front of a desk. In normal adults, the cerebral vascular response takes about 10–12 s to be completed (Wobst et al., 2001; Meek, 2002). Thus, we adjusted the IGT test to detect DLPFC activation associated to each choice: approximately 12 s were allowed between choosing cards. Specifically, the participant could make each choice only after the presentation of a green slide on a computer screen, which was positioned in front of him or her on the same table where the IGT was performed, behind the four card packs. The slide was presented for 6 s, then there was a break of other 6 s, when the computer screen turned black. Then, the green slide was shown again, for the successive IGT choice and so it went on for the 100 picks of the entire DM task (Figure 2). We hypothesized that, separating each choice from the following one by 12 s, it would allow us to assess the DLPFC activation associated with each choice (Schroeter et al., 2004), with a limited interference on the IGT learning processes (Gupta et al., 2009). The entire experimental procedure was completed in about 25 min.

### Data analysis

Our analyses focused on the increase of HbO2 concentration, which is considered an estimate of cerebral activation (e.g., Meek, 2002). Possible components of the HbO2 signal related to slow fluctuations of cerebral blood flow and heartbeat noise were removed by bandpass filtering between 0.02 and 1 Hz and, in order to prevent movement artifacts, a further filter was used to remove detections with rapid changes in HbO2 concentration (signal variations > 0.1 mM•mm over two consecutive samples). We also visually checked the signals recorded in each channel of all subjects, in order to detect low signal-to-noise ratio due to bad transmission of near-infrared light (e.g., due to hair obstruction). There was no need to exclude any registrations from data analysis due to a bad signal-to-noise ratio.

An event design was used and the active channels were identified by means of paired *t*-tests. In each participant, for every channel, an arbitrary baseline was calculated as the mean in relative changes of HbO2 in the 2 s before the onset of the green slide. The hemodynamic response associated with each choice was calculated as the mean change in HbO2 concentration over the 10 s after the onset. To identify the activated channels, e.g., those showing a HbO2 increase, we performed one-tailed paired *t*-tests and compared, for each channel, the baseline and the choice associated hemodynamic response. In order to evidence possible changes in DLPFC activation associated with different choice risk levels, as the performance went on, the time course of task execution was divided in four periods, each one consisting of 25 choices, and DLPFC activation was distinctly analyzed for low and high risk choices in each period.

To control Type I error in multiple testing situations, we used a false discovery rate (FDR) approach (Genovese et al., 2002; Singh and Dan, 2006), that controls the proportion of false positives among channels that are significantly detected. We selected a *q* value of 0.05, so that there were no more than 5% false positives (on average) in the number of channels emerging with significant contrasts. Statistical analyses were conducted using SPSS version 13.0 for Windows (SPSS Inc., Chicago, IL, USA).

## Results

All but one participant in our sample chose advantageously, showing an IGT net score above the cut-off threshold of at least 50 choices from advantageous decks. Table 2 reports single performances.

**Table 2:** **List of IGT participants' performances (in bold it is reported the one with a score under the cut-off threshold of at least 50 advantageous choices)**.

**Participant id**	**Sex**	**Age**	**Igt score**
1	M	26	76
2	F	24	58
3	F	25	56
**4**	**F**	**26**	**36**
5	M	27	84
6	M	35	56
7	F	35	78
8	F	26	86
9	M	21	78
10	F	38	66
11	F	22	62

**Figure 3:**
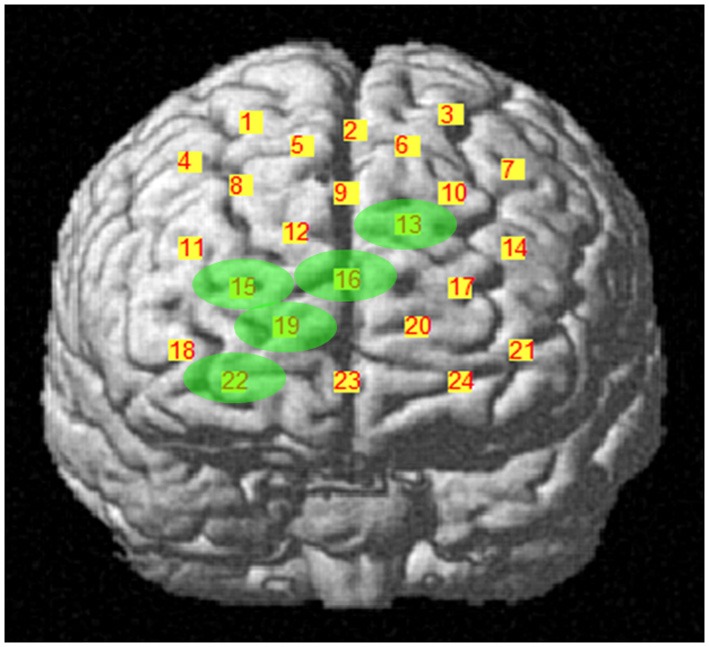
**Cortical location, on a rendered brain, of channels found significantly activated in association with low risk choices (evidence in green), during the first IGT period**.

**Figure 4:**
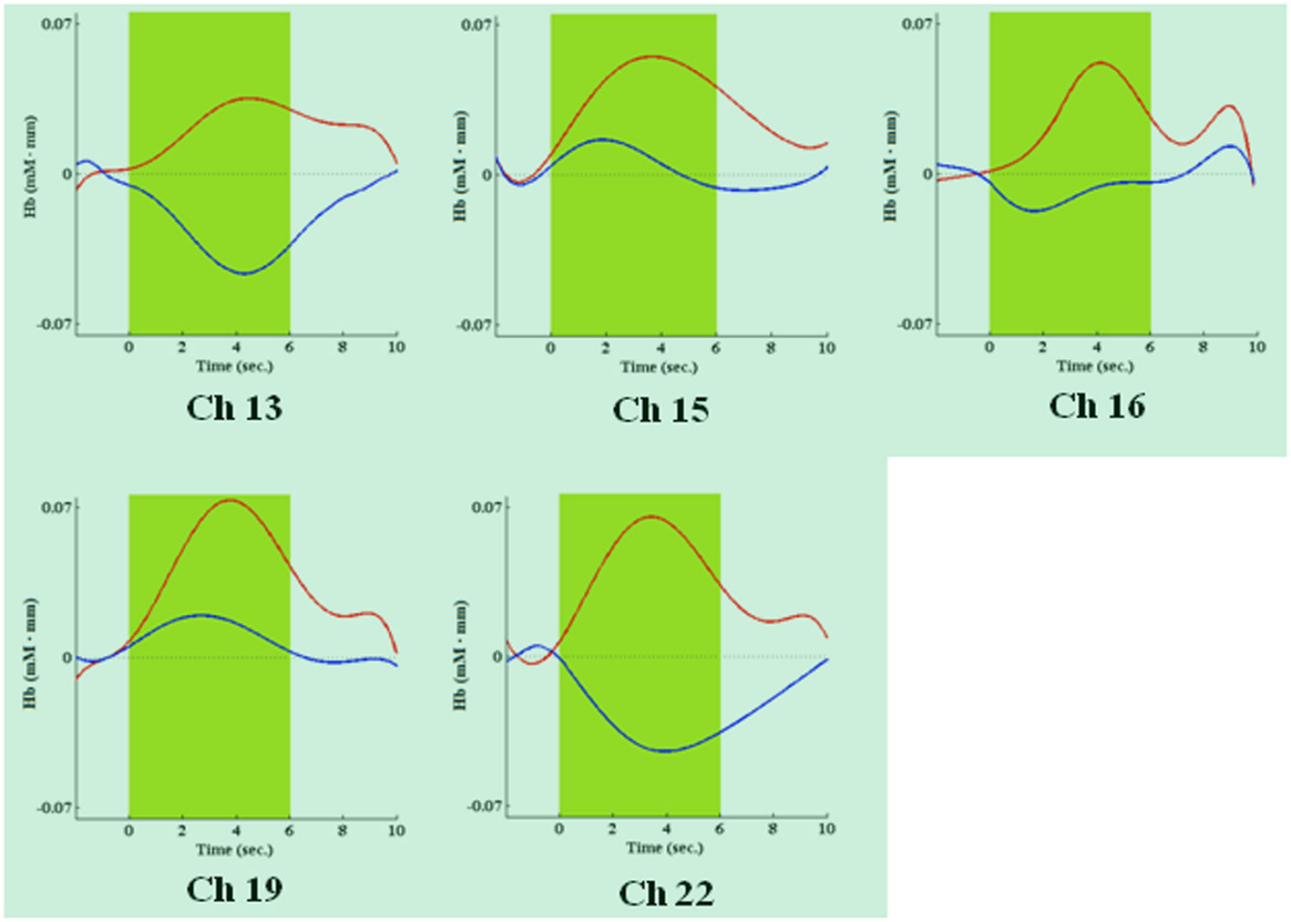
**Time courses of HbO2 (in red) and deoxy-hemoglobin (in blue) concentration changes, reported in mM•mm, in channels found significantly activated during the first IGT period, in association with low risk choices**. The dark green area shows when the green slide was presented on a computer screen to participants, signaling that the choice could be made.

During the first task period (1st–25th choice), five channels passed the FDR threshold (*P* < FDR 0.05) in association with low risk choices (advantageous card decks C and D). They were channel 13 (*t*_10_ = −2.818; *P* = 0.009), located on left superior frontal gyrus, channel 15 [*t*_(10)_ = −6.471; *P* < 0.001], located on right superior frontal gyrus, channel 16 [*t*_(10)_ = −3.454; *P* = 0.003], located on right/left superior and medial frontal gyrus, channel 19 [*t*_(10)_ = −3.042; *P* = 0.006], located on right superior frontal gyrus, and channel 22 [*t*_(10)_ = −3.222; *P* = 0.0045], located on right superior/orbital frontal gyrus (Figures 3, 4). Instead, in association with high risk choices (disadvantageous card decks A and B), no channel passed the FDR threshold.

**Figure 5:**
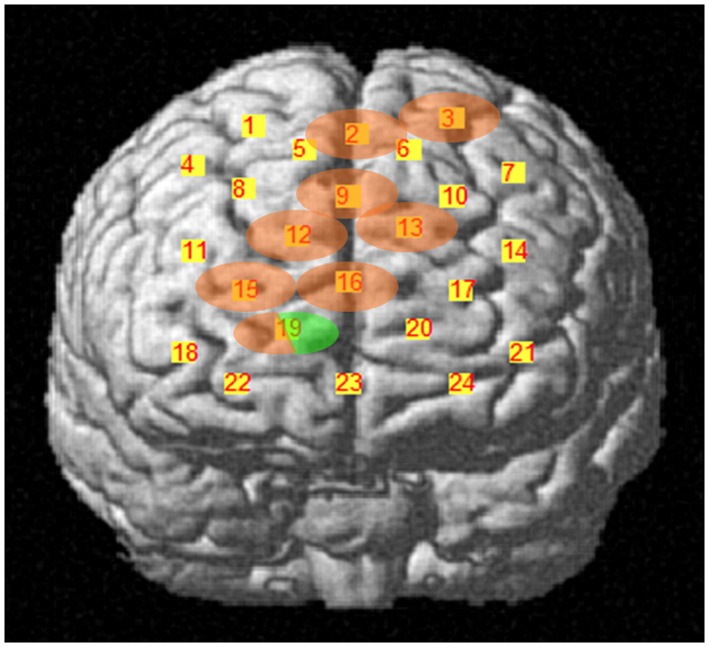
**Cortical location, on a rendered brain, of channels found significantly activated in association with high risk choices (evidence in pink), or in high and low risk choices (evidence in pink/green), during the second task period**.

**Figure 6:**
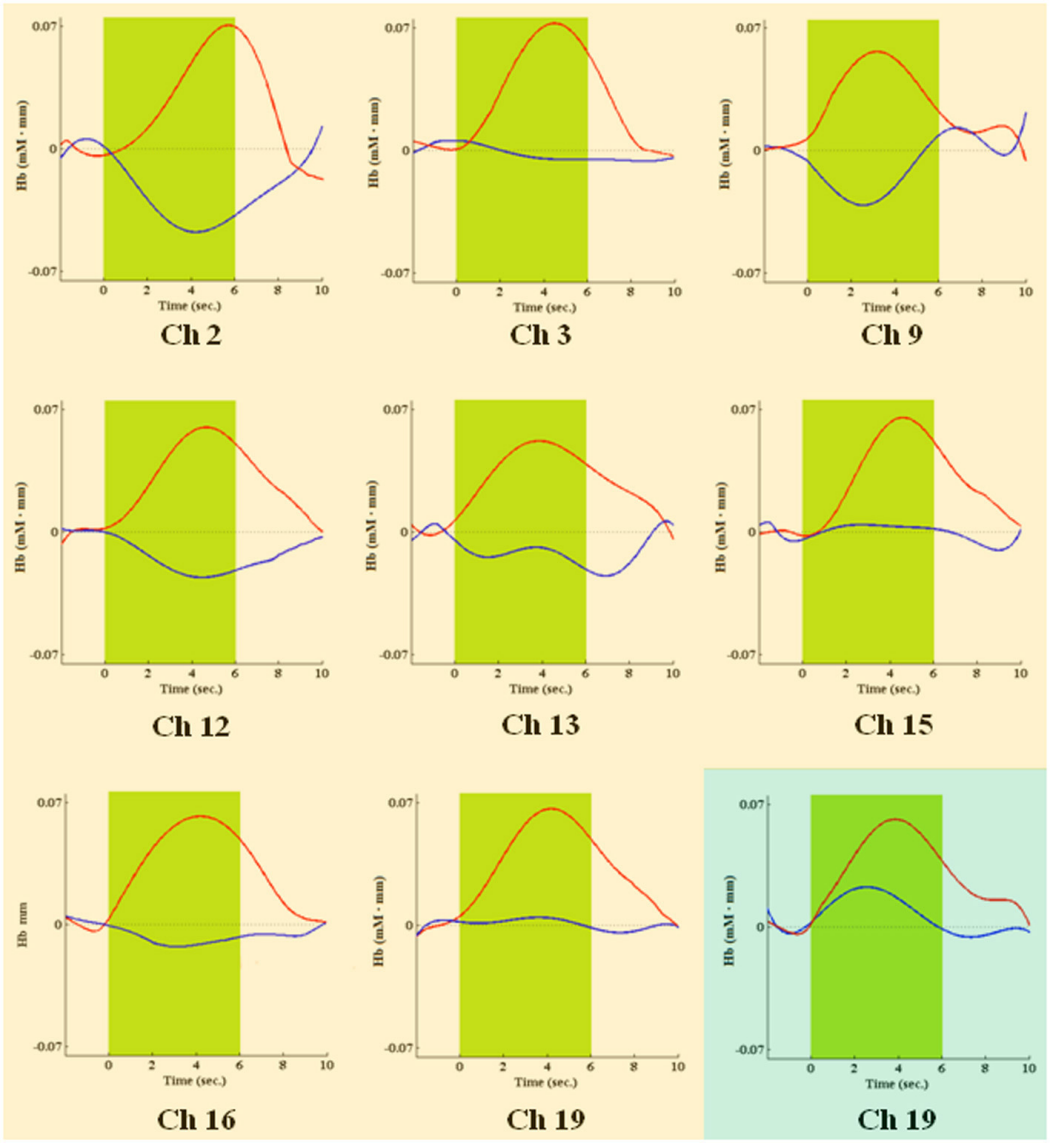
**Time courses of HbO2 (in red) and deoxy-hemoglobin (in blue) concentration changes, reported in mM•mm, in channels found significantly activated during the second IGT period, in association with high risk choices (pink background) or low risk choices (green background)**. The green area shows when the green slide was presented on a computer screen to participants, signaling that the choice could be made.

During the second task period (26th–50th choice), in association with low risk choices, only channel 19 [*t*_(10)_ = −3.910; *P* = 0.0015], located on right superior frontal gyrus, passed the FDR threshold. In association with high risk choices, on the other hand, eight channels passed the FDR threshold (*P* < FDR 0.05). They were channel 2 [*t*_(10)_ = −5.858; *P* = 0.006], located on right/left superior and medial frontal gyrus, channel 3 [*t*_(10)_ = −4.300; *P* = 0.001], located on left superior frontal gyrus, channel 9 [*t*_(10)_ = −3.841; *P* = 0.0015], located on right/left superior and medial frontal gyrus, channel 12 [*t*_(10)_ = −3.441; *P* = 0.003], located on right superior frontal gyrus, channel 13 [*t*_(10)_ = −3.349; *P* = 0.0035], located on left superior frontal gyrus, channel 15 [*t*_(10)_ = −4.078; *P* = 0.001], located on right superior frontal gyrus, channel 16 [*t*_(10)_ = −2.688; *P* = 0.0115], located on right/left superior and medial frontal gyrus, and channel 19 [*t*_(10)_ = −4.382; *P* = 0.0005], located on right superior frontal gyrus (Figures 5, 6).

During the third and fourth task period (51st–100th choice), no channel passed the FDR threshold (*P* < FDR 0.05), for either low- or high-risk choices.

Four channels were activated in association with both low- or high-risk choices. They were channels 13, 15, 16, and 19, covering left, right and central portions of DLPFC. Additionally, in association with low-risk choices, also the right frontal pole was activated (channel 22), while, in association with high-risk choices, the DLPFC activation extended more posteriorly, to channels 2, 3, and 9.

## Discussion

We used multichannel NIRS to study if DLPFC is differentially activated, over time, by low or high risk choices during the execution of the complete administration of IGT, a playing card task developed to study DM under uncertainty. We have found that DLPFC significantly contributes to the IGT execution in the first half of the task. Additionally, we found different time courses in its activation as the task went on, associated with low or high risk choices. Specifically, DLPFC was shown to be activated by low risk choices in the first period (1st–25th choice) and by high risk choices in the second period (26th–50th choice) of IGT performance. Activated DLPFC areas associated with low or high risk choices partially overlapped in both left, right and central portions of the DLPFC region monitored. In association with low risk choices (first period), along with activation of left/right superior frontal gyrus and left/right medial frontal gyrus (channels 13, 15, 16, and 19), also the right frontal pole was activated (channel 22). In association with high risk choices (second period), the DLPFC activation was more extensive and involved more posterior areas, possibly including the supplementary motor area (channels 2 and 3). In both cases, activation was slightly lateralized on the right side. No significant variations in DLPFC activity emerged during the second half of IGT execution, regardless of choice risk level.

The involvement of DLPFC, especially with a right lateralization, in IGT execution has already been shown. Clinically, a bad IGT performance was observed in patients with right DLPFC lesions (Clark et al., 2003; Fellows and Farah, 2005) and, by neuroimaging, DLPFC cortical areas were found activated during the IGT test (Ernst et al., 2002, 2003; Bolla et al., 2003, 2005). Besides DLPFC, other areas may play a role in DM, such as VMPFC (Bechara et al., 2000a), the amygdala (Bechara, 2001; Bar-On et al., 2003) and the insula (Lin et al., 2008; Lawrence et al., 2009), but such areas are too deeply located to be detected by NIRS devices. Primary and secondary sensory areas (Bechara, 2001; Bar-On et al., 2003) are involved in DM as well, but they were outside our region of interest. More recently, Hartstra et al. (2010) have demonstrated, by fMRI, that activation of DLPFC during IGT execution changes over time: it is significant in the first part of the modified task they used and becomes non-significant later. These authors speculate that earlier activation observed in DLPFC may be attributed to the involvement of working memory, which would be necessary for active learning of task rules (see also Bechara et al., 1998; Clark et al., 2003; Fellows and Farah, 2005; Martinez-Selva et al., 2006). In addition, they hypothesize that later med-OFC activation is associated with the representation of learned reward values, guiding successive choices. In agreement with the previous study, we showed an early significant involvement of DLPFC in IGT performance as well, but we found a differential activation according to choice risk level. DLPFC was activated earlier by low risk choices and later by high risk choices. Study designs differed, as Hartstra et al. (2010) aimed to study the role of prefrontal cortex in rules learning, while we aimed to show the DLPFC contribution to IGT performance in the setting of high or low risk choices. In this regard, a meta-analysis of fMRI studies has demonstrated that risk-related DLPFC activation occurs when a choice is involved (Mohr et al., 2010). Moreover, it has been recently found that DLPFC mediates the discrimination of IGT disadvantageous choices (Christakou et al., 2009), and the activation intensity in such region has been positively correlated with task performance (Lawrence et al., 2009).

Our results on the differential DLPFC activation over time, in association with low or high risk choices during the IGT, may seem to indicate that working memory is not the only neurocognitive resource supplied by DLPFC. We speculate that two superior cognitive functions, mediated by DLPFC as well, may explain our observations: attention shifting and response inhibition. It is worth noting that, in IGT, high risk choices are those with a greater immediate reward, but with a delayed greater loss. On the contrary, low risk choices are associated with lower immediate rewards, but with a delayed gain. DLPFC activation showed by our participants seems to indicate a shift in attention, as the task went on. Their attention shifted from the decks with lower immediate rewards, in the first task period (1st–25th choice), to those with greater losses, in the second task period (26th–50th choice). In both cases, an attention response, mediated by DLPFC (e.g., Manes et al., 2002), was elicited by those decks (and related choices) that seemed to be more disadvantageous (see also Christakou et al., 2009; Lawrence et al., 2009). Moreover, in the second period of the task, when DLPFC was activated in association with high risk choices, frontocentral cortical areas (channels 9 and 16) and the supplementary motor area (channels 2 and 3) were also activated. Both regions have been associated with response inhibition and behavioral control (Elliott et al., 2000; van Gaal et al., 2008; Fassbender et al., 2009).

Our experimental procedure aimed to assess DLPFC activation associated with low or high risk choices, as they were made. Consequently, the activation of cortical regions with a role in response inhibition was detected when the high-risk decision was made. We speculate that the extended DLPFC activation in the second IGT quarter, may reflect a conflict between previously learned and newer rules, in order to make the best decision. The activation involving the right frontal pole in the first quarter may indicate an attempt to elaborate more abstract task rules (e.g., Venkatraman and Huettel, 2012), as an initial approach to an unknown complex task. Concerning the absence of DLPFC activation in the third and fourth period of the IGT, we speculate that it may be due to VMPFC taking over the task, which is undetectable by fNIRS. Indirectly, this hypothesis is supported by Hartstra et al. (2010), who found only a later involvement of medial prefrontal cortex regions (med-OFC in their case) in IGT performance. Moreover, Lawrence et al. (2009) found a linear decrease in lateral OFC and pre-supplementary motor area activation as IGT execution progressed.

Our study has some obvious limitations. First, we enrolled a limited number of subjects and it should be considered a pilot study. Although functional neuroimaging studies are typically performed in small populations, our results need to be confirmed in a larger sample. Characterizing participants for personality traits and mood state would further improve such a study, since it has been proved that these variables have an influence on IGT performance (Buelow and Suhr, 2009). Second, multichannel NIRS has a poorer spatial resolution than fMRI. However, previous research has shown a good correlation between these two techniques (Toronov et al., 2001; Strangman et al., 2002). Third, NIRS measurement of haemoglobin variations are limited to the lateral surface of the cerebral cortex. Fourth, the experimental design we chose did not allow us to discriminate among the specific prefrontal functions involved in the early distinction between low- and high-risk choices. Finally, some possible systemic effects we have not directly checked, e.g., blood flow variation in the scalp during the experiment, could have had an influence on our results. However, this was probably not the case because an asymmetric pattern of cortical activation was observed.

In conclusion, this study has further deepened the complex role of DLPFC to early DM under uncertainty process, as assessed by IGT. We have shown that, in the first half of the performance, DLPFC activation differs over time, according to the risk level of the choice. We propose that, beside working memory, other complex functions, such as attention shifting and response inhibition, are involved in sustaining the DLPFC role in discriminating disadvantageous choices during the task. Thereafter, as rule learning establishes, DM under uncertainty seems to be prevalently mediated by VMPFC. Thus, it emerges a wide, complex and temporally dynamic involvement of the prefrontal cortex in decisions concerning the ordinary uncertainty of everyday life.

### Conflict of interest statement

The authors declare that the research was conducted in the absence of any commercial or financial relationships that could be construed as a potential conflict of interest.
